# Ethanol consumption in non-human primates alters plasma markers of bone turnover but not tibia architecture

**DOI:** 10.1038/s41598-024-65021-4

**Published:** 2024-06-19

**Authors:** Alibek Zhakubayev, Lara H. Sattgast, Anne D. Lewis, Kathleen A. Grant, Russell T. Turner, Urszula T. Iwaniec, Mary Lauren Benton

**Affiliations:** 1 Department of Computer Science, Baylor University, Waco, TX 76798 USA; 2 Skeletal Biology Laboratory, School of Nutrition and Public Health, Oregon State University, Corvallis, OR 97331 USA; 3 Division of Comparative Medicine, Oregon National Primate Research Center, Oregon Health and Science University, Beaverton, OR 97006 USA; 4 Division of Neuroscience, Oregon National Primate Research Center, Oregon Health and Science University, Beaverton, OR 97006 USA; 5 Center for Healthy Aging Research, Oregon State University, Corvallis, OR 97331 USA

**Keywords:** Bone architecture, Ethanol, Non-human primate, Bone, Bone quality and biomechanics, Risk factors

## Abstract

Ethanol consumption is associated with positive, negative, and neutral effects on the skeletal system. Our previous work using a nonhuman primate model of voluntary ethanol consumption showed that chronic ethanol use has an impact on skeletal attributes, most notably on biochemical markers of bone turnover. However, these studies were limited by small sample sizes and resulting lack of statistical power. Here, we applied a machine learning framework to integrate data from 155 monkeys (100 ethanol and 55 controls) to identify the bone features associated with chronic ethanol use. Specifically, we analyzed the influence of ethanol consumption on biomarkers of bone turnover and cancellous and cortical bone architecture in tibia. We hypothesized that chronic ethanol use for 6 months to 2.5 years would result in measurable changes to cancellous features and the biochemical markers compared to control animals. We observed a decrease in bone turnover in monkeys exposed to ethanol; however, we did not find that ethanol consumption resulted in measurable changes in bone architecture.

## Introduction

Alcohol is a bioactive nutrient that is consumed at varying levels worldwide^1^. Alcohol consumption impacts most organ systems, including the skeleton, where it has variable and incompletely understood effects. The complexity of its actions is associated with sex, age, drinking patterns, and comorbidities, and likely other, as yet undefined, factors. In humans, alcohol consumption is reported to have positive, neutral, or negative skeletal actions^2^. Due to ethical concerns, intervention studies in humans are uncommon and most studies have been observational. Consequently, animal models have played an important role in understanding the skeletal response to alcohol^2^.

The majority of animal studies investigating the effects of alcohol on bone have been performed in growing rodents. These studies have shown that chronic heavy alcohol consumption reduces bone accrual, leading to osteopenia. Several putative mechanisms have been identified. These include alcohol-induced increases in oxidative stress and impairment of growth hormone signaling^3,4^. However, most human alcohol consumers are adults and relatively few evaluations have been performed in skeletally mature (post linear and radial bone growth) rodents. Long duration (14-week) consumption of high levels of alcohol resulted in cancellous bone loss whereas moderate alcohol consumption lowered bone turnover without negatively impacting bone mass or architecture in skeletally mature rats^5,6^.

Rodent models have contributed to our understanding of the skeletal actions of alcohol but have important limitations. Small rodents have no secondary (Haversian) remodeling, the principal mechanism for remodeling cortical bone in adult humans. This is important because (1) cortical bone remodeling is essential to preserving bone quality, (2) cortical bone comprises approximately 80% of the total bone mass in humans, and (3) cortical bone is critical to the mechanical function of the skeleton^7^. To circumvent this limitation, we have investigated the skeletal response to alcohol in non-human primates. Non-human primates are an attractive model given their high genetic, physiological, and behavioral similarities to humans. The similarities across species include robust intracortical bone remodeling and drinking behavior^8^. To date, we have investigated the skeletal effects of ethanol consumption in two species of male and female macaques^9–13^. These studies revealed dose, age, sex, and species-associated differences in bone response to ethanol. However, the studies were limited by the small sample size of individual cohorts often resulting in insufficient statistical power to detect meaningful effects. The goal of the present analysis was to leverage data from multiple cohorts, totaling 155 monkeys, to identify the bone features associated with chronic ethanol use. All monkeys in this evaluation were subjected to a consistent and well-established ethanol self-administration protocol^14^. We hypothesized that extended heavy ethanol consumption would result in measurable changes in bone (tibial mass, density and/or architecture) and biochemical markers of bone turnover compared to controls.

## Results

### Bone features stratify species and sex, but not ethanol consumption

**Figure 1:**
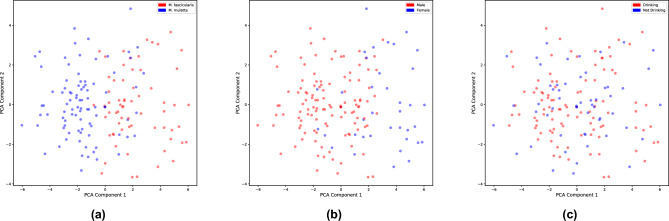
Individuals cluster by species and sex based on bone features, but not by ethanol consumption. The plots show separation between individual animals in PC1 (x-axis) and PC2 (y-axis) based on (**a**) species, (**b**) sex, or (**c**) ethanol consumption. Points are colored by group.

We applied Principal Component Analysis (PCA) to the cortical, cancellous, and biochemical features in our dataset to reduce the data to a smaller number of dimensions. After that, we visualized the data in the reduced space (PC1 vs. PC2; Fig. 1). We colored the points based on either species, sex, or ethanol consumption. The proportion of variance explained by the first two principal components is 0.496 and 0.211, respectively, totaling 70.7% of the variance explained.

In Fig. 1a, the data were colored based on species (blue points are rhesus macaque and red points are cynomolgus macaque). Due to known biological differences between the two species, such as size, we expected separation in the plot. For species, we observe a clear visual separation in the first principal component. The blue points are on the left side of the plot, while the red points are on the right. It is also well known that there are significant biological differences between males and females. In Fig. 1b, we colored the data based on sex. Similarly, we hypothesized that there would be a clear separation between males and females. In the plot, we see that the female monkeys are clustered mainly on the right side of the plot, while the male monkeys are on the left side. Finally, in Fig. 1c, we colored the data in the PCA space based on the drinking category. The red points represent monkeys who consumed ethanol in the experiment, while the blue points represent the control monkeys. In contrast with the previous plots, we do not observe a clear separation between the ethanol and control group.

**Table 1:** Significance of the difference between bone features when stratified by ethanol use and correlation between bone features and drinking status. Significant results ($p < 0.05$) are shown in bold.

Variable	Difference in means (p-value)	Correlation
Osteocalcin	7.97 (**3.0e**−**4**)	− **0.29**
CTX	0.23 (**2.1e**−**3**)	− **0.38**
Cross sectional volume	2.17 (0.54)	0.06
Cortical volume	0.76 (0.52)	0.01
Marrow volume	1.25 (0.36)	0.10
Cortical thickness	3.22 (0.44)	− 0.08
Polar moment of inertia (PMOI)	36.83 (0.41)	0.06
BMC	0.04 (0.74)	− 0.03
BMD	0.01 (0.15)	− 0.14
Area	0.44 (0.72)	0.04
Length	2.27 (0.26)	0.08
Bone volume/tissue volume (BV/TV)	0.62 (0.59)	− 0.05
Connectivity density	0.01 (0.36)	0.07
Trabecular number	0.01 (0.80)	0.01
Trabecular thickness	4.95 (0.32)	− 0.10
Trabecular separation	1.92 (0.65)	− 0.05

### Biochemical markers of bone turnover differ after ethanol consumption

Table 1 shows the difference between the ethanol and control groups in their cortical, cancellous, and biochemical features. We compared the mean of each feature between the two groups and reported the correlation between the feature and drinking status. From these tests, we can conclude that osteocalcin and C-terminal telopeptide (CTX) levels differ in drinking animals compared to controls, and that drinking ethanol is associated with a reduction in the values of both biomarkers. However, we do not observe a significant difference in other bone features between the ethanol and control animals.

To further explore the relationship between bone features and ethanol use, we trained a random forest model to classify drinking and control monkeys. Given the clustering in Fig. 1, we included known covariates such as species, sex, and age in our model. We then extracted the importance of each feature from the trained model to understand the strength of the relationship between the feature and the classification as drinking or control. Table 2 contains the feature importance values from the random forest; here, higher valued features have a greater influence on the model output. Age is the most important variable, followed by CTX and osteocalcin levels. This was replicated in a logistic regression model (Supplementary Table 1), where age (odds radio [OR] = 0.48, p-value = $6.6\times 10^{-6}$) and osteocalcin levels (OR = 0.91, p-value = $1.9\times 10^{-3}$) were associated with decreased odds of being in the drinking category. The polar moment of inertia was also significantly associated with increased odds of being in the drinking category; however, the effect was small (OR = 1.03, p-value = $3.3\times 10^{-3}$). Taken together, these results highlight that bone architecture is the least predictive of drinking status.

**Table 2:** Feature importance from the random forest model predicting drinking status.

Feature	Importance
Age	0.153
Sex	0.004
Species	0.003
CTX	0.091
Osteocalcin	0.098
Cortical volume	0.043
PMOI	0.040
Length	0.038
BV/TV	0.054
Trabecular separation	0.041
Marrow volume	0.052
Trabecular thickness	0.036
Cross sectional volume	0.044
Connectivity density	0.062
Area	0.038
BMD	0.064
Cortical thickness	0.035
Trabecular number	0.048
BMC	0.048

### Influence of ethanol on biomarkers shows similar trends across species and sex

In the previous sections, we showed the results using all available data. To address the generalizability of our results across species and sex, we quantified the association between ethanol use and bone features for animals stratified by species and sex.

Table 3 shows the p-values and correlations for each subgroup (i.e., species, sex) when comparing ethanol to control groups. We highlighted the significant p-values and the top two correlation values for each comparison. In rhesus macaques, the results were very similar to the results using the overall dataset (Table 1). Across all subgroups, osteocalcin is among the most highly correlated features, highlighting a negative relationship between ethanol use and osteocalcin level. For the males, marrow volume significantly differs between the control and drinking groups ($p = 0.04$). For cynomolgus macaques and females, there were no significant differences in any feature between drinking and control animals, although the direction of the correlations is consistent with the other subgroups and the full group analysis. This may be due to a smaller sample size compared to both the rhesus macaque and male groups.

**Table 3:** Significance of the difference between bone features and correlation between the feature and drinking status when stratified by both ethanol use and species/sex. Significant values ($p < 0.05$) and the top two correlations are shown in bold.

	*M. mulatta*		*M. fascicularis*		Male		Female	
	p-value	Corr	p-value	Corr	p-value	Corr	p-value	Corr
Osteocalcin	**8.80e**−**3**	− **0.31**	0.27	− **0.23**	**2.84e**−**4**	− **0.33**	0.38	− **0.16**
CTX	**2.57e**−**4**	− **0.49**	0.25	− 0.11	**1.79e**−**4**	− **0.49**	0.35	− 0.15
Cross sectional volume	0.70	− 0.05	0.64	0.04	0.13	0.21	0.43	− 0.14
Cortical volume	0.62	− 0.06	0.81	− 0.10	0.12	0.12	0.41	− 0.15
Marrow volume	0.74	− 0.04	0.22	0.22	**0.04**	0.23	0.30	− **0.19**
Cortical thickness	0.94	− 0.00	0.05	− **0.29**	0.69	− 0.04	0.57	− 0.10
PMOI	0.68	− 0.05	0.81	0.05	0.08	0.16	0.34	− 0.14
BMC	0.32	− 0.13	0.66	− 0.06	0.85	0.02	0.75	− 0.05
BMD	0.31	− 0.15	0.25	− 0.17	0.22	− 0.14	0.53	− 0.12
Area	0.50	− 0.08	0.93	0.01	0.29	0.12	0.91	0.02
Length	0.77	0.01	0.85	− 0.03	0.11	0.17	0.84	0.05
BV/TV	0.63	0.06	0.38	− 0.13	0.93	− 0.01	0.17	− 0.25
Connectivity density	0.99	0.03	0.18	0.10	0.34	0.09	0.98	0.00
Trabecular number	0.96	0.04	0.79	− 0.04	0.76	0.03	0.70	− 0.07
Trabecular thickness	0.83	0.02	0.23	− 0.18	0.50	− 0.07	0.47	− 0.13
Trabecular separation	0.66	− 0.05	0.71	− 0.05	0.82	− 0.07	0.77	0.05

### Animals show significantly different biomarker levels after drinking

We showed that osteocalcin and CTX values are two of the features most highly correlated with ethanol consumption. These biomarkers were collected for each animal before and after the open access period, so we compared the levels of osteocalcin, CTX, and the ratio of osteocalcin to CTX within the control and ethanol groups pre- and post-ethanol exposure (or experiment duration for controls).

As expected, the values of osteocalcin and CTX did not change significantly for the control group. The mean value of osteocalcin increased by 0.93 ng/ml in the control group, while it decreased in the ethanol group by 2.57 ng/ml. Similarly, there was a larger difference in CTX levels in the ethanol group (decrease of 0.43 ng/ml) compared to the control group (decrease of 0.21 ng/ml). The ratio of osteocalcin to CTX was significantly different before and after the experiment in both groups, suggesting that even small differences in individual biomarkers can have an influence on the balance of bone turnover. Table 4 shows the p-values of the statistical tests.

**Table 4:** Significance of the difference between osteocalcin and CTX levels and the ratio of osteocalcin to CTX pre- and post-experiment. Significant differences ($p< 0.05$) are shown in bold.

Group	Osteocalcin	CTX	Osteocalcin/CTX
Control	0.75	0.08	**0.03**
Ethanol	**0.01**	**7.58e−9**	**2.94e**−**4**

### Duration of ethanol exposure does not significantly predict bone features

In addition to species and sex, other extrinsic features such as duration of ethanol exposure could play a role in the level of skeletal response to ethanol. To better understand if these extrinsic features affect the cancellous, cortical, and biochemical features we trained a set of random forest regression models on drinking, species, sex, and experiment length. The target variables in these experiments were the cancellous, cortical, and biochemical measures. Table 5 shows the random forest regression model results for each bone feature. Under the drinking, species, sex, and experiment length columns, we show the feature importance. The $R^2$ column provides the performance of the regression model.

All regression models that perform well ($R^2 > 0.5$) do not include experiment length as an important feature. Therefore, we concluded that monkeys from groups with different experiment lengths can be compared since the feature has a limited effect on the model. The most important features for the successful random forest models were species and sex. This is consistent with our previous result that there are significant differences between the species and between sexes.

**Table 5:** Mean importance for experimental or demographic features predicting bone measures. Rows are sorted by the magnitude of the $R^2$.

Feature	Drinking	Species	Sex	Experiment length	$R^2$
Length	0.010	0.693	0.269	0.027	0.803
Cross sectional volume	0.016	0.654	0.302	0.028	0.822
Area	0.013	0.542	0.379	0.067	0.676
BMC	0.033	0.354	0.464	0.149	0.665
PMOI	0.016	0.638	0.322	0.025	0.704
Cortical volume	0.020	0.601	0.344	0.034	0.721
BMD	0.100	0.305	0.361	0.233	0.590
Marrow volume	0.035	0.532	0.343	0.089	0.596
Cortical thickness	0.098	0.269	0.308	0.325	0.164
Osteocalcin	0.284	0.272	0.109	0.335	0.173
Trabecular thickness	0.145	0.343	0.239	0.273	0.103
BV/TV	0.090	0.410	0.072	0.428	0.091
Trabecular number	0.221	0.157	0.246	0.376	0.243
Connectivity density	0.208	0.180	0.317	0.296	− 0.269
Trabecular separation	0.216	0.253	0.189	0.342	− 0.175
CTX	0.345	0.417	0.117	0.121	− 0.526

## Discussion

In this study, we used a machine learning framework to analyze the influence of ethanol consumption on (1) two established biomarkers of bone turnover (osteocalcin and CTX), and on (2) cancellous and cortical bone parameters from the tibia of non-human primates. In addition to evaluating the differences based on ethanol consumption, we also compared the bone features across sex and species (rhesus macaque vs. cynomolgus macaque).

Our statistical analyses showed that osteocalcin and CTX differed between drinking and non-drinking animals; however, the tests for all other features did not result in statistically significant differences. The feature importance measures calculated from our machine learning model also showed that osteocalcin and CTX are the features most affected by ethanol consumption. For example, in the control group we observed no significant differences over time, but in the group that consumed ethanol we found a significant reduction in osteocalcin and CTX after drinking.

We performed a similar analysis on the subgroups (male vs. female, rhesus vs. cynomolgus) to determine whether any of these trends differed by sex or species. We observed generally consistent trends in the bone features across the subgroups. The differences in osteocalcin and CTX levels before and after drinking were the most significant in each subgroup, with a direction of effect that was consistent with the full analysis. We note, however, that interpretation of these results is limited due to the small sample size in several of the subgroups (Table 6).

Most intervention studies evaluating the effects of alcohol on bone in animal models and humans have been single cohort studies with small group size^2^. Typically, they have been of short duration and have modeled a narrow range of drinking. The present analysis evaluated 13 cohorts of monkeys (totaling 155 monkeys; 55 control and 100 ethanol) exhibiting a wide range of drinking behavior (i.e., low, binge, chronic heavy). This range of drinking recapitulates human drinking behavior.

It is notable that ethanol had effects on biochemical markers of bone formation (osteocalcin) and resorption (CTX) independent of treatment duration, but no effect on tibia mass, density, or architecture. We interpret this as evidence that the rates of bone formation and bone resorption in tibia were each lowered by alcohol such that no net change in bone mass was detected. Moderate alcohol consumption (3–6% of caloric intake) similarly resulted in decreases in osteoblast- and osteoclast-lined bone perimeter with no bone loss in proximal tibia metaphysis of skeletally mature rats^6^. However, bone turnover rates and balance vary among bones and bone compartments. Factors such as baseline level of bone turnover and magnitude of mechanical loading can influence rates of bone loss^15^. In this regard, we detected a decrease in cancellous bone volume fraction in lumbar vertebra of male rhesus monkeys following 1 year of open access drinking^12^. The turnover rate in the region of interest was almost 100%/year which is much larger than the 4–6%/year rates observed on tibial bone surfaces (periosteal, endocortical, and intracortical).

Chronic alcohol abuse is an established risk factor for osteopenia and low trauma fracture. However, comorbidities known to negatively influence bone metabolism are common in individuals who abuse alcohol^2^. These include poor diet, smoking, cirrhosis, pancreatitis, and diabetes. Some, but not all, studies suggest that alcohol consumption has no independent effect on bone mineral density in men and women^16–19^. The present analysis in non-human primates supports the concept that heavy alcohol consumption by adults suppresses bone turnover^2^. In contrast to many chronic heavy alcohol consumers, the monkeys in our studies had a nutritionally balanced diet and appeared free of comorbidities. Consequently, the absence of change in bone architecture in monkeys consuming ethanol suggests that comorbidities contribute to the bone loss often observed in human chronic alcohol consumers.

This analysis has several limitations. The bone mass, density and architecture measurements were performed on a single bone. This may be important because bone remodeling rates differ among bones as well as skeletal compartments. It will be important to extend this analysis to include additional bones. A strength of the analysis is that we evaluated both sexes from two species of monkeys. However, the number of animals in drinking and non-drinking groups varied, as well as the number of monkeys across sex and species, which could limit our ability to detect group differences. Finally, the resolution of the $\mu$CT, while sufficient to accurately quantify bone dimensions and important architectural parameters such as cortical bone thickness, and cancellous trabecular number, thickness and separation, is insufficient to measure Haversian canal number and size. This is a significant limitation because studies performed using a more sensitive analysis in three cohorts of male rhesus monkeys suggest that alcohol reduces cortical bone porosity^13^. Additional studies should be performed to confirm the porosity results.

In summary, this analysis does not support our hypothesis that long duration alcohol consumption would result in measurable changes in bone (tibial mass, density and/or architecture). However, it does provide evidence that, independent of sex and species, alcohol consumption lowers bone turnover in adult monkeys. This finding is important because chronic low bone turnover may negatively impact bone quality.

## Methods

### Study population

The current study uses data derived from an ongoing multidisciplinary project investigating ethanol addiction and end organ effects of ethanol in non-human primates. We analyzed bone samples from two species of macaques: *Macaca mulatta* (rhesus macaque) and *Macaca fascicularis* (cynomolgus macaque). We used the cancellous and cortical bone features collected from the tibia along with blood biomarkers to examine the differences between sex, species, and drinking categories. Table 6 describes the distribution of our population. We included a total of 155 monkeys in the study. Across all cohorts, there were 66 cynomolgus macaques and 89 rhesus macaques; of these, there were 55 control animals and 100 drinking animals. Archived tissue and data from all individuals are available in the Monkey Alcohol Tissue Research Resource (MATRR)^20^. The studies were approved by the Institutional Animal Care and Use Committee at the Oregon National Primate Research Center and complied with ARRIVE guidelines. The study was conducted in accordance with relevant guidelines and regulations.

All animals used in this study adhered to a well-established schedule-induced polydipsia (SIP) protocol as described in Grant et al.^14^. Briefly, animals were induced to drink 4% ethanol w/v in water by delivering a 1-g banana-flavored pellet every 5 min (Noyes, Lancaster, NH; Bio Serv, Flemington, NJ). Following a 30-day water induction period, the monkeys consumed 0.5 g/kg/day ethanol, followed by 1.0 g/kg/day, and, finally, 1.5 g/kg/day. Each ethanol dose was consumed for 30 consecutive days. The escalating volumes of ethanol ensured that each animal experienced elevated ethanol levels as measured by blood ethanol concentrations (BECs), and the volume equivalent to the 1.5 g/kg/day dose ensured that most animals experienced intoxication, as defined by a BEC of 80 mg/dl. After ethanol induction, the monkeys were allowed open access to 4% ethanol w/v for 22-h each day for variable length of time ranging from 6 months to 2+ years, depending on the cohort.

In this study, we split the monkeys into a drinking and a control group. The drinking group had open access to ethanol (4% w/v) for at least 6 months. Bones were collected after 179–911 days. The control group did not have open access to ethanol. Instead, they were given an isocaloric volume of maltose-dextrin solution during the open access period. Due to the standardization of the protocol, data can be integrated across cohorts. The relative timing of events, including the blood draws for biomarker assays, is consistent across the cohorts.

**Table 6:** Summary of cohort information for animals analyzed in this study. Cohort number refers to the cohort from the MATRR. Experiment length is measured in days.

Species	Group	Sex	Cohort #	Exp. length	Number of monkeys
Rhesus macaque	Drinking	Male	4	413	10
		Male	5	363	8
		Female	6a	336	6
		Female	6b	344	5
		Male	7a	358	8
		Male	7b	348	5
		Male	10	694	8
		Male	14	599	9
	Control	Male	5	363	5
		Female	6a	336	3
		Female	6b	344	3
		Male	7a	358	4
		Male	7b	348	4
		Male	10	694	8
		Male	14	599	3
Total					**89**
Cynomolgus macaque	Drinking	Male	2	911	11
		Female	3	444	10
		Female	8	179	3
		Male	9	204	8
		Male	13	212	9
	Control	Male	2	911	11
		Female	3	444	4
		Female	8	179	4
		Male	9	204	3
		Male	13	212	3
Total					**66**
Overall total					**155**

### Biomarkers of bone turnover

Blood samples were collected during the week prior to the induction period and at necropsy. Samples collected prior to necropsy were obtained within the first 3 h of the 11-h light cycle as previously described^21^. Plasma was stored at $-80 \; ^\circ$C until analysis. Osteocalcin and CTX were measured by the Endocrine Technologies Core at the ONPRC using a Roche Cobas e411 Automated Clinical Platform (Roche Diagnostics, Indianapolis, IN). The ranges of the osteocalcin and CTX assays were 0.5–300 ng/ml and 0.01–6.00 ng/ml, respectively. Intra-assay coefficient of variation (CV) for osteocalcin was 7.8% and intra-assay CV for CTX was 1.1%. Because all samples were measured the same day, no inter-assay CV was calculated for these specimens.

### Dual-energy X-ray absorptiometry

Bone area (cm$^2$), bone mineral content (BMC, g), and areal BMD (g/cm$^2$) were determined ex vivo in the right tibiae with fibula attached using dual-energy X-ray absorptiometry (DXA) (Hologic Discovery A, Waltham, MA; Hologic APEX System Software, Version 3.1.1).

### Microcomputed tomography

**Figure 2:**
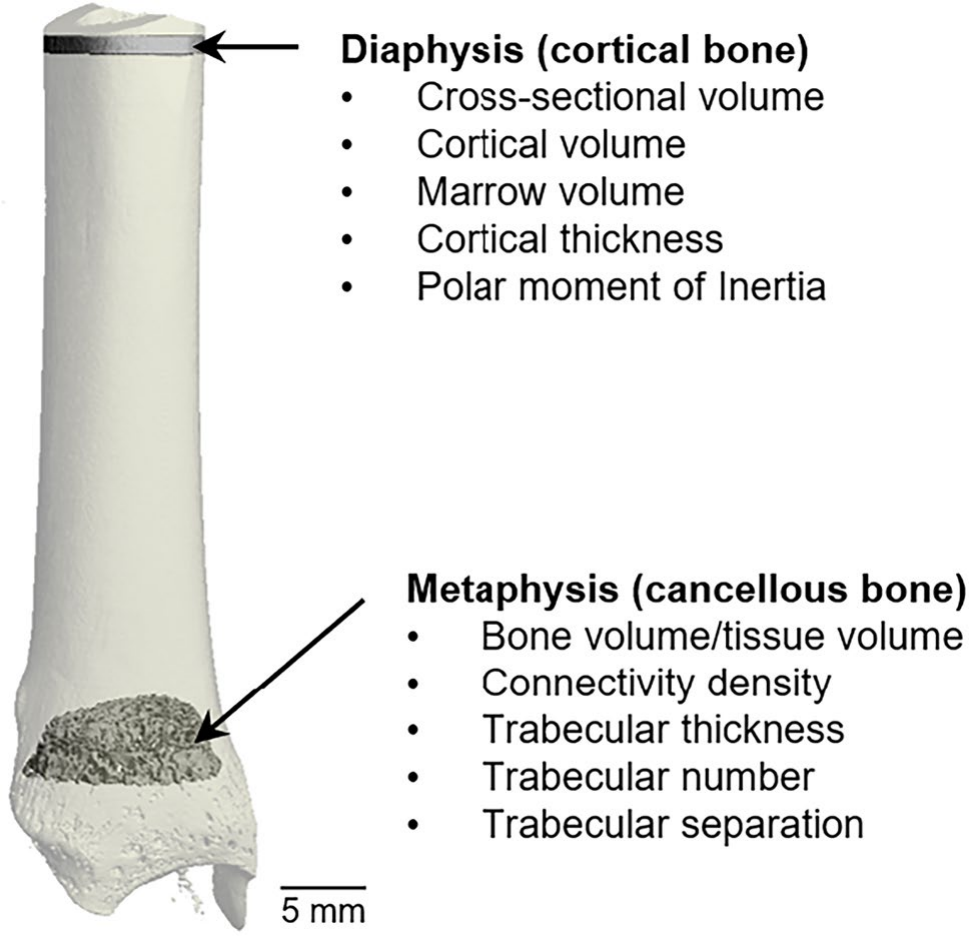
Regions of interest, including features, evaluated in the distal tibia diaphysis (cortical bone) and distal tibia metaphysis (cancellous bone).

Microcomputed tomography ($\mu CT$) was used for nondestructive 3-dimensional evaluation of cortical and cancellous bone architecture in the distal tibia. Tibial length was measured as the distance between the proximal tip of the intercondylar eminence and the distal tip of the medial malleolus. The distal third of the tibia was then excised using an IsoMet®Low Speed Saw (Buehler, Lake Bluff, IL) and scanned in 70% ethanol at a voxel size of 30 $\times$ 30 $\times$ 30 $\upmu$m (55 kVp, 145 $\upmu$A, and 200 $\upmu$s, 500 projections/rotation) on a Scanco $\mu CT40$ scanner (Scanco Medical AG, Basserdorf, Switzerland). Evaluations were conducted with filtering parameters sigma and support set to 0.8 and 1, respectively. The regions of interest evaluated are shown in Fig. 2.

Thirty-three consecutive slices (990 $\upmu$m) of cortical bone (at proximal end of the distal third of the tibia) were analyzed at a threshold of 245 (grayscale of 0–1000) determined empirically. Cortical measurements included cross-sectional tissue volume (cortical and marrow volume, mm$^3$), cortical volume (mm$^3$), marrow volume (mm$^3$), cortical thickness ($\upmu$m), and polar moment of inertia (an estimate of bone strength in torsion, mm$^4$). In addition, 66 consecutive slices (1980 $\upmu$m) of bone were evaluated in the distal tibia metaphysis at a distance of 165 slices (4950 $\upmu$m) proximal to the caudal growth plate at a threshold of 175 (gray scale of 0–1000) determined empirically. Cancellous bone measurements included cancellous bone volume fraction (bone volume/tissue volume %), connectivity density (mm$^{-3}$), trabecular thickness ($\upmu$m), trabecular number (mm$^{-1}$), and trabecular separation ($\upmu$m).

### Experimental design

We stratified the monkeys into one main comparison group based on ethanol consumption (drinking vs. control), and two subgroups for species (rhesus vs. cynomolgus) and sex (male vs. female). Our overall goal was to determine whether the bone features separated the monkeys into drinking and control animals; however, because ethanol has the potential to have different effects based on species or sex we also conducted subgroup analyses. We ran the first analysis on the entire dataset without any additional stratification, before conducting four additional analyses to evaluate each the following groups independently: (1) rhesus macaques, (2) cynomolgus macaques, (3) males, and (4) females.

We used data collected at baseline and after the open access period (or equivalent duration for controls). At baseline, we collected only biochemical features (osteocalcin and CTX). After the open access period, we collected both biochemical features and architectural features from the cortical and cancellous bones. Table 2 contains a list of all included features.

#### Data processing

To account for missingness and the differing ranges of our features, we imputed and normalized our data. In total, less than 3% of the data were missing. We imputed the missing data using mean imputation. We then normalized our data to ensure that all data points had a mean value of zero and a standard deviation of one.

#### Statistical analysis

We used the Student’s t-test to evaluate the difference in mean values between the drinking and control groups. We considered significance at a level of p $\le$ 0.05. Prior to performing the t-test, we assessed the normality of the data using the Shapiro Wilk test and evaluated the homogeneity of variances using Levene’s test. If either of the previous tests failed, indicating that the assumption for the t-test does not hold, we applied the Mann-Whitney U (MWU) test which does not make any assumptions about the distribution and variance of the data.

We calculated the Pearson correlation to quantify the linear relationship between group (e.g., ethanol vs. control) for each of the bone features. We note that a high correlation does not prove that drinking affects the tested bone features; we use this statistic as a complement to the t-test or the MWU. We applied PCA to reduce the dimensionality of our dataset and visualize the data.

#### Regression analysis

We conducted a logistic regression analysis to classify the animal into drinking/control based on the bone features and covariates such as species, sex, and age. We performed feature selection to remove the highly correlated predictors from our final model. Experiment duration could also influence the results of our analysis. Although most of the animals spent 12 months in the open access period, some cohorts had shorter (6 months) or longer (> 2 years) time in open access. To better understand the influence of experiment length, drinking, species, and sex on the bone features, we regressed each of these variables on our bone features using a random forest regression. We evaluated the coefficient of determination ($R^{2}$) for our model as well as the feature importance to understand which features, if any, were good predictors for our target data.

### Feature importance

We used machine learning to rank the strength of relationship between ethanol consumption and bone features (from cancellous bone, cortical bone, and biochemical markers). In this study, we applied a random forest classification algorithm. The random forest constructs an ensemble of decision trees, which are each trained on a random sample of data and a random set of features. The algorithm then aggregates predictions across all of the trees to produce the final output. This technique helps increase model generalization and decrease the chances of overfitting.

We trained our random forest model on the dataset of bone features, then reviewed the importance of each feature calculated by the model. The feature importance indicates the contribution of the feature to the prediction. Our random forest classification model focused on bone features as the input and ethanol consumption as the target to reveal which aspects of the skeletal system are most associated with ethanol consumption in our study.

### Supplementary Information

Supplementary Table 1.

## Data Availability

Data from all individuals analyzed in this study are available in the Monkey Alcohol Tissue Research Resource (MATRR), matrr.com.
