# Supplementary material for: Ethanol consumption in non-human primates alters plasma markers of bone turnover but not tibia architecture
Source: Sci Rep. 2024 Jun 19;14:14137. doi: 10.1038/s41598-024-65021-4 (PMC11187174; doi:10.1038/s41598-024-65021-4)
Supplement: Supplementary file 1 — Supplementary Table 1. [file 41598_2024_65021_MOESM1_ESM.pdf]

Supplemental Information

Table 1. Odds ratios from logistic regression model predicting drinking category.

Feature	Odds ratio of drinking (95% CI)	p-value
Species	0.14 (0.01-1.59)	0.12
Sex	0.53 (0.07-4.20)	0.54
Age	0.48 (0.34-0.64)	6.6E-6
Length	1.06 (0.96-1.18)	0.26
Area	1.11 (0.57-2.27)	0.77
BMC	0.26 (0.04-1.58)	0.15
Cortical volume	0.57 (0.31-0.96)	0.05
Marrow volume	0.81 (0.58-1.03)	0.15
Cortical thickness	1.00 (0.99-1.01)	0.48
PMOI	1.03 (1.01-1.05)	3.3E-3
BV/TV	1.01 (0.75-1.35)	0.94
Connectivity density	0.66 (0.35-1.19)	0.17
Trabecular thickness	1.00 (0.95-1.05)	0.98
Trabecular separation	1.00 (0.98-1.02)	0.84
Osteocalcin	0.91 (0.85-0.96)	1.9E-3
CTX	1.92 (0.49-8.70)	0.37
